# Beyond Morality: Assessment of the Capacity of Faith-based Organizations (FBOS) in Responding to the HIV/AIDS Challenge in Southeastern Nigeria

**Published:** 2018-01

**Authors:** Eze Edlyne ANUGWOM, Kenechukwu ANUGWOM

**Affiliations:** 1. Faculty of Arts, Dept. of Anthropology and Sociology, University of the Western Cape, Bellville, South Africa; 2. Faculty of Social Sciences, Dept. of Social Works, University of Nigeria, Nsukka, Nigeria

**Keywords:** HIV/AIDS, Faith-based organizations, Response, Capacity, Nigeria

## Abstract

**Background:**

For the world can get rid of the HIV/AIDS pandemic by 2030, there is need for more to be done especially in the case of countries in Africa. In Nigeria, such efforts have included Faith-Based Organizations (FBOs) recognized as partners in the National Response Framework. However, the extent to which these FBOs contribute to efforts to control the pandemic will depend on their capacity. Therefore, this study aimed to ascertain the technical and managerial capacity of these FBOs to respond to the pandemic in Nigeria.

**Methods:**

We utilized social survey in examining the capacity of three purposively selected FBOs in the Southeast of Nigeria to respond to the pandemic. Thus, the focus group discussion and the key informant interviews were used. The data for the study was collected between Feb and Apr 2014.

**Results:**

The study discovered a general low capacity but high willingness of the FBOs to get involved. One of the FBOs studied was better placed than others and had even established committee on the pandemic. However, in another FBO, the pandemic was still seen largely with moral lens that blame those infected rather than provide support. All the FBOs were ambivalent on the use of condoms as a prevention method.

**Conclusion:**

There is need for sustained capacity building for the FBOs in order to provide them with knowledge on the pandemic and help them act out the role envisaged for them in the National Response Framework in Nigeria.

## Introduction

In spite of the optimism of the UNAIDS, that the world can get rid of the HIV/AIDS pandemic in 2030 (1), a lot still needs to be done especially in the case of countries in Sub-Saharan Africa where in addition to bearing the highest burden of the affliction, nations are equally struggling with other high disease burdens and a declining public health provisioning. As the latest UNAIDS Factsheet shows, 36.7 million people are living with HIV globally; while 2.1 million people became newly infected with the virus in 2015 (2). Nearer home, the UNAIDS reports that 6.5 million people are living with HIV in Western and Central Africa. Equally, the UNAIDS reports that 9% of the people living with the virus globally are in Nigeria. In other words, the agency estimates that about 3.2 million people were living with the virus in Nigeria by 2014 (3). Despite this number, the National AIDS Control Agency (NACA) shows that there has been significant increase in the number of people in Nigeria now on active antiretroviral therapy (AART) in order to increase the survival of these seropositive individuals (4).

Since it was discovered, HIV/AIDS has become one of the most important development concerns globally. This concern is without doubt primary in the developing world especially in Africa where the disease can be considered the most potent challenge to the development of the continent. UNAIDS had earlier on reported that the epidemic has overtaken malaria as the traditional killer disease in Africa, home also to about 95% of all AIDS orphans in the world (5).

The impact of the epidemic as obvious from the above facts has necessitated a broad-based approach that involves different institutions. The Faith-Based Organisations (FBOs) have been identified in the National Framework on HIV/AIDS as one of the state level institutions in the fight against HIV/AIDS. Without doubt, the FBOs have played significant roles in the African response to the HIV/AIDS pandemic. These roles which have often gone against the grain of ideal medical strategies against the pandemic have all the same remained among the health seeking alternatives for those affected and inflicted by the pandemic. The niche occupied by the FBOs is perhaps highlighted by the fact that they can rightly be seen as providing a significant percentage of health services in Sub-Saharan Africa. Thus, the FBOs have been very critical in the mitigation, care and treatment of HIV/AIDS in Africa (6–11). This is in spite of the fact that such services usually intertwined with the spiritual often create tensions and go against perceived best medical practices. In other words, their overall response has often been dogged by controversy and even rejection especially as they reject condom use and promotion and refuse to accept the reality of sex outside marriage as part of the response (9).

Socio-cultural practices which have refused change have been also related to HIV/AIDS risk in Nigeria (12). One of the ways of countering the adverse influence of socio-cultural practices (apart from enlightenment and sensitization) especially in rural and urban illiterate populations is through Western religion which has become a way of life for over 80% of Nigerians in the Southern parts of the country. Hence, the influence of religion in people’s way of life in a country like Nigeria cannot be underestimated since it even becomes a crucial issue in the election of the president of the country (13, 14). This influence of religion makes it a critical tool in any campaign for behaviour modification among the general populace.

There is a certain ambivalence regarding both the ideal role and even the involvement of the FBOs in the HIV/AIDS response continuum (15, 16, 8). Despite the fact that quite a good number of research have been produced on the role of FBOs in the fight against HIV/AIDS, there is hardly any on the specific context of the Southeastern Nigeria where FBOs are dominant institutions overtly influential of health seeking behavior of the people. Therefore, this paper was an attempt to address the above gap in knowledge.

## Methods

The area of the study was Enugu state in Southeastern zone of Nigeria seen as typifying any other state in geo-political Nigeria and generally considered the capital and epicenter of the Southeast zone. Apart from a vibrant public service and commercial sectors, the state has a robust tertiary education sector with over five tertiary institutions. This large number of tertiary institutions in the state explains the high number of young people there. Enugu state in the sentinel survey in 2012 has an HIV prevalence rate of 4.1% to 6.0% in the general population (4).

There are over 350 registered FBOs or churches in the state (17). Out of this number, three FBOs that reflect the main distinctions in the Christian community in the area viz. the orthodox Christian churches (the Anglican Communion-Diocesan Action Committee on AIDS (DACA); the early Pentecostal churches (The Assemblies of God Church); and the African-American denominations (The Methodist Church) were purposively selected for the study. The purposive sampling was based on criteria of typicality in representing the three major Christian denominations and ease of access for the study. Some of the FBOs would rather not discuss HIV/AIDS. The study adopted the Focus Group Discussion (FGD) and the Key Informant Interviews (KIIs) as qualitative techniques for assessing the capacity of the FBOs to respond to the pandemic. While the KII targeted key informants especially HIV and AIDS focal persons/coordinators in the FBOs; three FGD sessions were conducted with the three FBOs; each session was made up of six members including male and female participants. The instruments were administered to the different organizations at their offices and other locations chosen by them. The data for the study were collected between Feb and Apr 2014. The study was motivated by the need to identify capacity-building needs of FBOs that were or could be engaged in the HIV/AIDS response. It was part of a health project management handbook being developed by the researchers.

## Results

### General Capacity and Readiness to Respond

The FBOs presented a mixed bag of capacity or response-ability. In general, they have focused essentially on prevention and awareness campaigns. While the Diocesan Action Committee on AIDS (DACA) can be seen as having gone beyond the average response level or systematically achieving especially in terms of having well-established structures and officers in charge of HIV/AIDS; another like the Assemblies of God is at the primary level of response and so have not established HIV/AIDS response structures. However, the Methodist Church was somewhere between the above two groups i.e. it has been able to identify the need for the response and has taken action though a systematic structure has not yet emerged. All the same, all of them had huge capacity gaps particularly in terms of partnership, networking, and linkages. As would be expected, the parochial nature of these organizations which often even when not voiced portrays the afflicted persons as wayward has negatively affected and limited their response.

The study examined the these FBOs in terms of issues of governance, experience and skill, partnership and service delivery seen in the National Response Framework as critical to the ability of any FBO to contribute meaningfully to the response. The key findings are presented below:

1. *Governance*
  - The Assemblies of God Church: The church members and the board are interested in the involvement of the church in the HIV/AIDS response. However, there is no obvious attempt to achieve gender equity in the membership of the board and there is no clear-cut policy that should inform the HIV/AIDS response of the church. In spite of this, there is the likelihood of high-level participation of the church members if well-coordinated.
  - DACA: The church has a relatively better structure since there is in existence a working committee on HIV/AIDS with experience and which enjoys the support of the top echelon of the church. However, the committee needs to be strengthened and this would improve its effectiveness. There is also need to take opportunity offered by the support of the church hierarchy to formulate a policy on HIV/AIDS response. In fact, the participants in the FGD felt that this could be easily achieved even though they were of the view that the church would need technical support from the government AIDS control agency in order to succeed with this.
  - The Methodist Church: The church has a constitution that even though good enough for regular church matters does not address HIV/AIDS; hence the governance of such activities is mainly on ad-hoc basis as directed by the leadership. However, the FGD participants and KII respondents felt that it was time for a more structured and focused approach. They were also particular about the need to strengthen and expand the social ministry of the church to take on board HIV/AIDS matters. They also agitated for increase in sermons that deal with the realities of the pandemic especially among young people.
2. *Partnership*
  - The Assemblies of God Church: The participants claimed that the FBO has collaborated with other religious groups and even the state AIDS agency in efforts in the past three years. However, in spite of this claim, there was evidently no established partnership on HIV/AIDS. Therefore, the participants agreed that the FBO seriously needs technical support and collaboration with other NGOs as well as linkages with external donors.
  - DACA: The consensus here was that the committee or DACA enjoys the commitment of members and had even included PLWHA as DACA members. It also needs to seek for more partners especially in terms of technical and financial support. In the words of one of the KII respondents, “there is so much that needs to be done and the church does not have inexhaustible resources. Apart from this, there is need for more training and even workshops”.
  - The Methodist Church: The results of the FGD showed that this FBO is still at the starting stage in terms of forging critical partnerships needed for effective response. However, the participants saw the commitment of members of the social committee as very high and that this could be harnessed to improve the overall capacity of the church to respond to the pandemic. The participants advocated for technical support and collaboration with other NGOs. They saw whatever exists in this regard now as haphazard and not formalized in any way.
3. *Experience and Skills of the FBOs*
  - Assemblies of God Church: The participants agreed that in terms of the required skills and experience to respond to the pandemic the church has nothing significant. In this sense, apart from the natural ability of the church to provide care and compassion to all those members who are sick (not limited to HIV/AIDS), there is no HIV/AIDS specific skill set or experience. In fact, the members were mainly in denial arguing that this situation may be a product of the fact that none of them can recall any member afflicted by the pandemic recently. In the words of one of them, “if such people exist then it must be that we are not aware of their real sickness. Of course, we have some sick members now and then and even some have been sick for long. What we have done is prepare and try to do the things that government says we need to do in terms of this pandemic”. However, in the course of the KII, a very senior member of the church conceded that such people might exist in the church and that “the only problem is that people are hardly bold to claim such status. Even when people want to access ARV, they often cover themselves or try to use proxies”.
  - DACA: Unlike the above, the participants here were of the opinion that the FBO has experienced, committed and skilled volunteer members of DACA. They also expressed the fact that the DACA was involved in organizing step down training on HIV/AIDS for different parishes under the diocese. They, however, agreed that there was need to build capacity of members of DACA on basic knowledge about HIV/AIDS and skills acquisition especially in the areas of treatment and referral.
  - Methodist Church: The participants in the FGD here were of the consensus that some members are experienced and knowledgeable about the pandemic and that even some of them serve as resource persons for other religious organizations and NGOs. They saw the need now for the inclusion of HIV/AIDS response as a broad portfolio in the activities of the social ministry of the church. In addition, they were of the opinion that the FBO needs to engage experts to build the capacity of members of the social ministry as well as help the church to organize step down training on HIV AIDS
4. *Service Delivery*
  - Assemblies of God Church: Unsurprisingly, the participants in the FGD here could not point to any evidentiary show of service delivery in terms of HIV/AIDS. Apart from reference to general cases of coming to the support and help of members who were sick, they could not delineate where such support was for HIV/AIDS specifically. However, it is likely that some of those who have been sick for long amongst their members may have become afflicted with the pandemic and are either unaware of the nature of their ailment or are not bold enough to come out openly with their ailment.
  - DACA: The participants here started off by enumerating the factors which position DACA as a reliable provider of services including the support of the Bishop and top echelon of the church; the multidisciplinary composition of DACA; and commitment of DACA members. The group went on to say that, the DACA is currently engaged in activities that can bring succor to PLWHA members and building capacity on health service delivery especially the opportunistic infections associated with HIV/AIDS.
  - Methodist Church: The FBO is seen as having established care and support program in reference to the pandemic and has engaged in activities that can bring succor to PLWHA. It has equally increased its preaching and exhortations against lifestyles that expose people to the affliction. Interestingly, the FGD participants knew about members suspected of being afflicted by the pandemic. However, the church should be supported by government and its agencies to do more. In collaborating this, a respondent in the KII averred that while it is “good to get the churches involved in the fight against AIDS in Nigeria which is like a recognition of the importance of religion, the government needs to now enable the church through providing resources and training to do this well”.

## Discussion

What emerges from the above is that the DACA comes out first in terms of responding to the pandemic; it is followed closely by the Methodist Church. However, the Assemblies of God Church has nothing concrete in relation to experience/skills and even cannot show evidence-based service delivery on HIV/AIDS. The FBO is mainly still engaged in the denial and self-righteous mode of the late 1980s and 1990s that blame people for getting infected rather than providing support. However, the church in spite of this conservatism recognizes both the overwhelming reality of the pandemic and the need for its response. The above difference in response and response readiness calls attention to the need for nuanced program on capacity building for FBOs, which factors in the different levels of capacity acquired by each of them.

There is also need as evident from the above findings for the FBOs to rise beyond seeing the response as part of traditional church charity. The churches generally perceive and respond to the pandemic mainly as one more issue of charity. This perception was very clear in the FGD sessions in all the three FBOs. However, very representative of this view is the opinion of a middle-aged female participant in the Methodist Church thus, “the church is a place where people come and receive charity. The church cannot stop its charity works even when it is in the case of this deadly affliction. This church always reached out to people in need and that is why we are very close here”. This is unhelpful since the HIV/AIDS pandemic demands the emergence of well-informed and skilled people in order to ensure that even FBOs add value to the national response.

In addition, there was glaring lack of knowledge even amongst some key members of the HIV/AIDS committees. In other words, there is need for training and the enhancement of the capacity of the FBOs if they are to fulfill the aim of including them as partners in the national response framework in Nigeria. In spite of acknowledged reality of the pandemic, some of the FGD participants found it difficult to talk at length about it. Even in some cases, energy was dissipated trying to establish either that the pandemic is rare among adherents or that those afflicted are new members who drifted in from other congregations. As one of the respondents in the KII contended, “we cannot close our doors to people even when they come with such sickness, we are a church. Most times, they would not even reveal the real nature of their aliment until much later”.

In spite of the FBOs being short on treatment and referral; they were critical both directly and indirectly (through membership exhortation on the need to take care of the afflicted) in the provision of home-based care (HBC) for those afflicted and in materially supporting those affected by the pandemic who are members of their congregation. The above findings reverberate with similar findings in the case of South Africa (7). In the words of a KII participant (key member of the DACA), “Christianity demands that we care for the sick. In fact, Jesus used this as example of Christian virtue when he asked, when I was sick did you take care of me. In as much as one frowns on such afflictions especially among married people, we are still Christians called to be our brother’s keepers”. Despite the above, there is a reassuring willingness and even commitment to get involved by these FBOs. Thus, on the query on whether the FBO wants to become more involved in the response, all the respondents answered strongly in the affirmative. They even went further to implore the researchers to intercede with the state AIDS control agency to get them more rigorously involved in its activities apart from the occasional workshop now and then. Given their massive numerical strength and acknowledged roles in health-seeking behavior of the people, it may be futile to exclude them from the response to the pandemic (18).

## Conclusion

In spite of the relatively impressive strides of one of the FBOs studied (DACA) in terms of capacity to respond, there is no doubt that the involvement of these FBOs in the response is fraught with challenges bordering on their parochial liturgical stance and rejection of some of the fundamental options in the national response i.e. the advocacy and promotion of condom use seen in another study as affecting their effectiveness (8). Therefore, there is need for robust engagement with these FBOs in order to build their capacity overtime and improve the skills and knowledge of members directly involved in the response. The goals of the National HIV/AIDS Response Framework can only be achieved in this manner.

## Ethical considerations

Ethical issues (Including plagiarism, informed consent, misconduct, data fabrication and/or falsification, double publication and/or submission, redundancy, etc.) have been completely observed by the authors.
